# The students' voice: Strengths and weaknesses of an undergraduate medical curriculum in a developing country, a qualitative study

**DOI:** 10.1186/1756-0500-4-256

**Published:** 2011-07-22

**Authors:** Priyanga Ranasinghe, Sashimali A Wickramasinghe, Ruwan Wickramasinghe, Asela Olupeliyawa, Indika Karunathilaka

**Affiliations:** 1 Faculty of Medicine, University of Colombo, Sri Lanka

## Abstract

**Background:**

In medical education, feedback from students' is essential in course evaluation and development. Students at Faculty of Medicine, University of Colombo, Sri Lanka complete a five year medical curriculum comprising of five different streams. We aimed to evaluate the five year medical curriculum at the Faculty of Medicine, University of Colombo, Sri Lanka.

**Methods:**

A qualitative research was conducted among recent graduates of the faculty. Students' opinions on strengths and weaknesses of the curriculum were collected via questionnaires, which were analysed and classified into common themes. A focus group discussion (FGD) based on these themes was conducted among two student groups, each comprising of a facilitator, two observers and nine students selected as a representative sample from questionnaire respondents. FGDs were conducted using a semi-structured set of open-ended questions to guide participants and maintain consistency between groups. The FGD evaluated the reasons behind students' perceptions, attitudes, emotions and perceived solution. Verbal and non-verbal responses were transcribed and analysed.

**Results:**

Questionnaire response rate was 82% (153/186). Students highlighted 68 and 135 different responses on strengths and weaknesses respectively. After analysis of both questionnaire and FGD results the following themes emerged: a well organized module system, increased frequency of assessments, a good variety in clinical appointments, lack of specific objectives and assessments at clinical appointments, community and behavioural sciences streams beneficial but too much time allocation, lengthy duration of course, inadequate knowledge provided on pharmacology and pathology.

**Conclusion:**

We demonstrate how a brief qualitative method could be efficiently used to evaluate a curriculum spanning a considerable length of time. This method provided an insight into the students' attitudes and perceptions of the present faculty curriculum. Qualitative feedback from students highlighted certain key areas that need attention and also possible solutions as perceived by the students'.

## Background

Curriculum evaluation can be broadly defined as the "continuous systematic process of gathering information about all elements of a curriculum, analysis and interpretation to help arrive at an understanding of the extent to which goals, objectives and outcomes have been achieved and subsequently take informed decisions for further improvement" [1]. Hence, systematic evaluation forms a cornerstone in curriculum development as it; helps to define the quality of educational experiences, demonstrates whether or not a program is meeting its educational goals and objectives, elicits feedback and satisfaction data from learners, helps to identify the need for changes thereby implementing improvements for future learners and enables medical educators to use evaluation data to disseminate evidence based educational innovations through presentations and publications [2].

Curriculum evaluation is a continuous process with a wide variety of different approaches. Characteristics of an ideal evaluation strategy are; reliability, validity, acceptability and inexpensiveness [3]. Medical students are often viewed as a reliable and valid source of information in curriculum evaluation as they observe teaching daily, in addition they are also a relatively inexpensive yet valuable resource. Students' descriptive evaluation of a curriculum forms an important instrument in curriculum development [3,4]. Structured questionnaire surveys are the most common evaluation tool presently used, however the quality and reproducibility of the data depends to a large extent upon the validity of the study instrument. In addition lengthy questionnaires may be viewed by students as a waste of their time, reducing the reliability of the data [3]. Focus group discussions are another valid evaluation process, often used in combination with other quantitative and qualitative methods [5].

**Figure 1:**
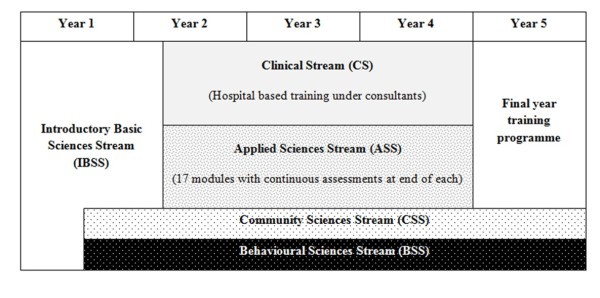
**Schematic representation of the five year curriculum at Faculty of Medicine, University of Colombo, Sri Lanka**.

The Faculty of Medicine, University of Colombo, Sri Lanka established in 1870 as the Colombo Medical School is the second oldest medical school in South Asia [6]. The Faculty at present provides for the education and training of undergraduates in allied health sciences. The medical undergraduate curriculum spans five year, the initial one and a half to two years of pre-clinical training is followed by three years of clinical training. The curriculum is subdivided into five main streams, initially starting from the Introductory Basic Sciences Stream (IBSS), followed by Applied Sciences Stream (ASS) and Clinical Stream (CS). The Community Sciences Stream (CSS) and Behavioural Sciences Stream (BSS) continue throughout the five year curriculum. The IBSS spanning the first one and half to two years focuses on teaching the basic sciences of Anatomy, Physiology, Biochemistry, Pathology, Microbiology, Parasitology and Pharmacology. The ASS comprises of 17 different modules based on different organ systems (Cardiovascular; Respiratory; Gastro-intestinal; Endocrine and Metabolism; Neurology; Musculoskeletal; Haematology and Immunology; Medico-legal; Genito-urinary; Infectious diseases; and etc), while the CS is mainly focused on clinical skills training and patient oriented hospital teaching. The ASS and CS teaching activities are conducted simultaneously, with students engaging in clinical activities at hospitals during daytime and ASS lectures during the afternoons. The students are divided into 'clinical' groups of 12-14 students each for clinical attachments during the CS. A diagrammatic representation of the present curriculum is given in figure 1. The present study evaluates the five year medical curriculum of the Faculty of Medicine, University of Colombo, Sri Lanka based on qualitative feedback from recent graduates.

## Methods

### Study population

A qualitative research was conducted among recent graduates of the Faculty of Medicine, University of Colombo, Sri Lanka for a period of two weeks in November 2008. One hundred and eighty six medical students graduating in year 2008 were invited for the study. Ethical clearance was obtained from the Ethics Review Committee, Faculty of Medicine, University of Colombo.

### Study instrument

Preliminary data were collected via a questionnaire, where each student was asked to list a minimum of five strengths and weaknesses, considering the entire five year undergraduate curriculum at the faculty. The emerging qualitative data were analysed by two members of the study team independently for emergent themes, later the two sets of themes were collectively classified into a single set of common emergent themes after an iterative consensus process among all members of the study team. Personal data of study participants were not collected and strict confidentiality was ensured.

### Focus group discussion

A focus group discussion (FGD) based on the themes emerging from the questionnaire was conducted among two different student groups. Each group comprised of a facilitator, two independent observers and nine students. The eighteen students for the two focus groups were selected randomly from the initial questionnaire respondents and proportionately matched for the distributions of gender, ethnicity and final undergraduate grading (Pass/Second class, lower division/Second class, upper division/First class) to the initial cohort of questionnaire respondents. A semi-structured set of open-ended questions for the FGDs were developed from emergent themes to guide participants and to maintain consistency between the two groups. The primary objective of the FGD was to evaluate the reasons behind students' perception, their attitudes and emotions, perceived solutions to overcome obstacles and strengthen the present curriculum. The facilitators were medical educationalists from the faculty; they provided guidance, maintained focus, stimulated constructive debate, regulated the flow of discussion and ensured time adherence, while maintaining a neutral stance on contents of discussion. Prior permission was obtained from each group for voice recording of the discussion. The two observers in each focus group independently transcribed verbal and non-verbal responses of the students. The verbal responses were transcribed in participants own words, while emotional responses of individuals and the entire group to different questions were also transcribed.

### Data analysis

Data obtained from the strengths and weaknesses questionnaire and the FGDs were analysed separately. The facilitator and two observers of each focus group were entrusted with the task of providing an analysis of verbal and non-verbal responses of participants in their respective groups. The final report is based on the questionnaire responses and the cumulative analysis of FGD results.

## Results

One hundred and fifty three students responded to the strengths and weaknesses questionnaire, of which 82 were males (53.6%). A high response rate of ≥82% was observed among all students, males and females. Sample characteristics are summarized in Table 1. Students highlighted 68 and 135 different responses on strengths and weaknesses respectively. After analysis of data, five themes were consistently expressed by a majority of students and were present in both focus groups. These five themes were considered as the 'major' themes. Three themes were deemed to be 'minor' as they were not consistently identified in both focus groups. The major emerging themes after the analysis of both questionnaire and FGD results were the following:

**Table 1:** Summarized characteristics of study participants

	Number of students (%)	
	
	Questionnaire respondents	FGD participants
Gender		
Male	82 (53.6%)	10 (55.6%)
Female	71 (46.4%)	8 (44.4%)

Ethnicity		
Sinhalese	136 (88.8%)	16 (88.8%)
Tamil	9 (5.9%)	1 (5.6%)
Muslim	8 (5.2%)	1 (5.6%)

Final MBBS result		
Repeat	16 (10.5%)	2 (11.1%)
Simple pass	83 (54.2%)	10 (55.5%)
Second class, Lower	40 (26.1%)	4 (22.2%)
Second class, Upper	12 (7.8%)	1 (5.6%)
First class	2 (1.3%)	1 (5.6%)

FGD - Focus Group Discussion

1. Inadequate knowledge provided on pharmacology and pathology during IBSS.

2. Structured and well-organized module system of the ASS.

3. Wide variety of clinical attachments during CS.

4. Inefficient community and behavioural sciences stream time allocation.

5. Lengthy duration and poor organization of entire course.

### Summary of major themes

#### 1. Inadequate knowledge provided on pharmacology and pathology during IBSS

The students felt that the IBSS was the most difficult time period at the faculty, as they were exposed to a vast amount of knowledge in seven subjects (Anatomy, Physiology, Biochemistry, Pathology, Pharmacology, Microbiology and Parasitology) over a relatively short period of time. Most of the subject matter was taught without emphasizing on the relevance to clinical application, and exam questions were mostly theory-oriented than clinical-oriented.

"We were not taught the clinical relevance of most of the subject matter in IBSS resulting in our studying being focused only towards passing exams with poor retention of clinically oriented subject-matter, which resulted in a substandard performance during clinical attachments"

This was mainly with regards to Anatomy and Biochemistry. The teaching activities in Physiology were seen as being more clinically oriented than the other six subjects and hence students' felt that it was an easier subject to study. Students' strongly felt that this irrelevant teaching in certain subjects compromised the teaching activities in Pathology and Pharmacology. In addition since Pathology and Pharmacology were taught during IBSS and the examination was a single 'combined' paper including all four subjects (together with Parasitology and Microbiology), more time, effort and attention was focused towards Anatomy, Biochemistry and Physiology.

"Due to inadequate teaching of Pathology and Pharmacology, we were at a disadvantage during clinical attachments and out inability to derive explanations to clinical scenarios from these two fundamental basic sciences were criticized frequently by clinicians during clinical attachments"

"Pathology and Pharmacology were taught during the ASS with separate modules, however since the individual module examinations in ASS were mainly clinical oriented we were able to pass exams without studying the Pathology and Pharmacology sections in each module"

The initial IBSS time period was also felt as being difficult due to several other reasons; a) it being a period of adjustment from teacher-based learning in schools to self-learning in the faculty, b) difficulties in language adjustment from 'Sinhala/Tamil' (native language) based school teaching to English based university teaching, c) inadequate guidance provided on study methods and recommended books, d) difficulties in adjusting to the university lifestyle and e) living separated from family members and loved ones.

#### 2. Structured and well-organized module system of the ASS

The students felt that the module based system during the ASS as a whole was well-organized and gave a broader picture on clinical oriented learning. However, the individual module structures were different from one another and certain important modules such as the Cardiovascular system module were poorly organized. They strongly agreed that the module workload was comparatively less than the IBSS. In addition having frequent assessments at end of each module was seen as an advantage as it helped to maintain focus on studies. Sometimes modules were not well aligned with the clinical appointments that the students' were engaged in during the same time period.

"For most of us the motivation for learning was having exams and hence frequent exams during the ASS was very helpful"

"We completed the Neurology clinical attachment more than 8 months prior to the Neurology module during the ASS and if we had the relevant module teaching prior to the clinical attachment it would have been better"

Module lectures by clinicians were very efficient and helpful. Students felt that having no vacation period during the lengthy ASS was stressful. Students were strongly dissatisfied with the frequent changes to module lecture schedules.

"Each module concluded with a short study leave and exam, immediately followed by the commencement of the next module"

"When lecturers have other commitments there is frequent alteration in the lecture schedule, which should not happen"

#### 3. Wide variety of clinical attachments during CS

The wide variety of clinical attachments during the CS was seen as being advantageous and clinical attachments significantly helped to retain studied knowledge. However teaching during individual clinical attachments varied immensely between different clinical groups and depended entirely on the in-charge consultant/specialist. This resulted partly due to inefficient interactions between the faculty staff organizing the curriculum and clinicians in charge of clinical attachments at hospitals.

"Objectives of each clinical attachment were not specific and they were poorly communicated to consultants/specialist in-charge of clinical attachments"

"Consultants/specialist should be given a basic training on teaching, as the variation in ability to teach among different specialist compromised our clinical knowledge"

The students' expressed their concern about not having a patient-oriented assessment at the conclusion of most clinical attachment. The viva-voca at the end or Gynaecology and Obstetrics clinical attachment and OSCE at the conclusion of Paediatric clinical attachment was seen as advantageous.

"Having an assessment at the end of each clinical attachment would help to lessen the performance anxiety during the final year patient oriented clinical examinations"

The time allocations for certain clinical attachments (Urology, Neurosurgery and Orthopaedics) were seen as being inadequate. In additions the clinical/hospital teaching environment was seen as more threatening than the faculty teaching environment.

#### 4. Inefficient community and behavioural sciences stream time allocation

The students strongly felt that the final year compulsory CSS and BSS teaching activities placed a significant burden on final year clinical studies and compromised performance. In addition having the final CSS and BSS examinations during the study leave for the competitive final MBBS examination was seen as being disadvantageous. They felt that the teaching activities and examinations of CSS and BSS should conclude prior to the commencement of the final year. They suggested that CSS and BSS learning activities be condensed into individual modules and completed during a short period, rather than spreading over the entire curriculum. However, both subjects were seen as important in-spite of the inefficient time allocations.

"The CSS and BSS teaching gives us an added advantage over students from other medical faculties in the country both at undergraduate and postgraduate level, however there is potential for the teaching activities to be more efficiently organized"

#### 5. Lengthy duration and poor organization of entire course

The students expressed their strong dissatisfaction about the avoidable lengthy duration of the faculty curriculum. This resulted from lack of proper organization and coordination between various departments.

"We were initially told that the duration of the curriculum would be four and a half to five years, however it took exactly six years to complete"

"There were long time periods (gaps) in the curriculum without any teaching learning activities for most students"

### Summary of minor themes

Problem based learning (PBL), activities during various topics in the curriculum was seen as inefficient due to several reason;

"We were not able to grasp the concept of PBLs as it is a new concept to our faculty, and hence it failed to serve the expected purpose"

"Most facilitator were not properly trained on conducting PBLs, while PBLs conducted by experienced facilitators were very useful"

Having no ragging (a verbal, physical or psychological abuse on newcomers to educational institutions by senior students'; prohibited at the faculty) and no student union clashes leading to disruption of teaching activities was seen as an advantage when comparing to the other medical faculties in the country.

Students expressed concern about not having proper training on writing answers to Structured Essay Questions (SEQs).

"We were not given a proper training on writing SEQ answers as done in many other medical faculties in the country, hence we were at a disadvantage"

Students also felt that the faculty should take steps to initiate a training programme and assessment of clinical examiners as there was a large variation between examiners at clinical exams. The answers to MCQ papers not being discussed with students after completion of exams was seen as a disadvantage as it is immensely important for learning and correcting mistakes.

## Discussion

To our knowledge this is the first study from Sri Lanka evaluating a medical curriculum spanning five years. Several other studies have focused either on specific educational interventions [7,8] or evaluated only a specific section of the curriculum [9]. Curriculum evaluation in many ways drives the development and evolution of curricula [10]. Evaluation can have a formative role, identifying areas where teaching can be improved, or a summative role by judging the effectiveness of teaching. Studies have demonstrated that in medical curriculum development, students' descriptive evaluations are helpful in forming a learner-centred knowledge-building process [4].

In this first comprehensive evaluation of the five year curriculum at Faculty of Medicine, University of Colombo we demonstrate how brief qualitative methods could be effectively used to evaluate a curriculum that spans a length of time. The study highlights the perceptions of recent graduates who have completed all components of the five year teaching programme and their suggestions for potential improvements. The recent graduates having completed all components of the curriculum were able to provide critical feedback on positive and negative aspects of each curriculum component as reflected by the results of our evaluation. Feedback from the graduates has been accepted by most teachers as valid and reliable, hence proved to be a useful tool to stimulate curriculum change [11]. The relatively high response rate observed in the present study indicates a higher acceptability of the method by questionnaire respondents. The other main advantages of this method were the manageability and cost effectiveness of the process and the ability to create an environment enabling students to express their views freely on curricular matters. However a potential limitation is the smaller sample sizes as some of the students' views could be biased. Similar studies evaluating medical curricula from India [12] and Pakistan [13] have used a questionnaire based approach. Although questionnaires are the most commonly used method of curriculum evaluation, it was considered inappropriate in the present context where all components of the curriculum were evaluated. A lengthy questionnaire may have been viewed by students as a waste of their time, reducing the reliability of the data [3]. Questionnaire based evaluations are better suited for evaluation of educational interventions or a specific sections of curricula.

Focus group interviews have been used quite frequently in health services program planning, medical education, and curriculum planning [5]. In the present study, where we evaluated all components of the five year curriculum the focus group discussions ensured a quick and reliable method of evaluation over a short period of time, whilst enabling us to obtain a large volume of data. The data obtained from the strengths and weaknesses questionnaire were helpful in guiding the focus group discussion. The main purpose of an evaluation is to guide curriculum development as no curriculum is perfect in design and delivery. Hence it is important that the results of evaluation to correct deficiencies are acted on and contents are updated. The constructive feedback provided by recently graduated medical students of Faculty of Medicine, University of Colombo, Sri Lanka were used to effect changes to the present faculty curriculum, for example the subjects Pathology, Pharmacology, Microbiology and Parasitology were moved from the IBSS section of the curriculum and taught as a separate module ('Foundation' module) soon after the completion of the IBSS, thus placing due emphasis on these subjects while achieving a better integration amongst the subjects. Hence the IBSS section of the 'modified' curriculum consists only of the three subjects Anatomy, Physiology and Biochemistry. However, curriculum evaluation and development is a continuous process and it is important to regularly assess the impact of these changes in view of further improvement. Our findings also enables further focused qualitative or quantitative research in to the areas of the curriculum identified as being deficient.

The present study has several limitations, the use of medical educationists as facilitators of the focus groups had the potential to prohibit students from critiquing aspects of the course and the topic that they may have otherwise done with a facilitator not associated with the course, Faculty or University. Despite this potential for bias, students commented during the focus groups that the presence of the course coordinator did not influence their responses. In addition there is potential interviewer bias because researchers, who were fellow faculty, conducted the focus group discussions. To counter this potential bias, we had members of the study team who were not academic staff members of the faculty to analyse questionnaire responses and prepare the semi-structured set of focus group discussion questions.

## Conclusions

This brief qualitative assessment provided a useful insight into students' attitude and perspectives of the present day medical curriculum at the Faculty of Medicine, University of Colombo, Sri Lanka. Qualitative feedback of the students on the curriculum highlighted certain key areas that need to be given attention and also possible solutions to overcome these deficiencies.

## Abbreviations

IBSS: Introductory Basic Sciences Stream; CSS: Community Sciences Stream; BSS: Behavioural Sciences Stream; ASS: Applied Sciences Stream; CS: Clinical Stream.

## Competing interests

The authors declare that they have no competing interests.

## Authors' contributions

PR, IK, AO and SAW made substantial contribution to conception and study design. SAW and WMRPTB were involved in data collection. AO and PR were involved in refining the study design, statistical analysis and drafting the manuscript. WMRPTB, PR and IK critically revised the manuscript. All authors read and approved the final manuscript

## Authors' information

**Ranasinghe P: **Is a MBBS graduate at the Faculty of Medicine, University of Colombo. Has served as a Research Associate at the Department of Clinical Medicine, Faculty of Medicine, Colombo, Sri Lanka. His research interests include Medical Education and Diabetes Mellitus.

**Wickramasinghe SA: **Is a MBBS graduate at the Faculty of Medicine, University of Colombo. She has served as a Research Assistant at the Medical Education Development and Research Centre (MEDARC), Faculty of Medicine, Colombo, Sri Lanka.

**Wickramasinghe WMRPTB: **Is a MBBS graduate at the Faculty of Medicine, University of Colombo. He has served as the President of the Medical Students' Union at the Faculty of Medicine, University of Colombo, Sri Lanka. His research interests include Medical Education

**Olupeliyawa A: **Is a MBBS graduate at the Faculty of Medicine, University of Colombo. He is a junior educationalist and researcher who serves as a Lecturer in Medical Education while following his postgraduate studies in medical education. He has several publications in medical education.

**Karunathilaka I: **Is the Director of the Medical Education Development and Research Centre (MEDARC), Faculty of Medicine, Colombo, Sri Lanka. He received training in Medical Education at the Centre for Medical Education, University of Dundee. He has authored many publications in medical education and has been a resource person at national and international forums.
